# Music Therapy for Patients Who Have Undergone Hematopoietic Stem Cell Transplant

**DOI:** 10.1155/2014/742941

**Published:** 2014-01-09

**Authors:** Chelsea G. Ratcliff, Sarah Prinsloo, Michael Richardson, Laura Baynham-Fletcher, Richard Lee, Alejandro Chaoul, Marlene Z. Cohen, Marcos de Lima, Lorenzo Cohen

**Affiliations:** ^1^Department of Psychology, University of Houston, Houston, TX 77008, USA; ^2^Department of General Oncology and the Integrative Medicine Program, The University of Texas MD Anderson Cancer Center, 1400 Holcombe, Unit 462, Houston, TX 77030, USA; ^3^College of Nursing, The University of Nebraska Medical Center, Room 5071, 985330 Nebraska Medical Center, Omaha, NE 68198-5330, USA; ^4^Department of Medicine-Hematology and Oncology, University Hospitals Seidman Cancer Center, Case Western Reserve University, 11100 Euclid Avenue, Cleveland, OH 44106, USA

## Abstract

*Objectives*. This study examines the short- and long-term QOL benefits of a music therapy intervention for patients recovering from hematopoietic stem cell transplantation (HSCT). *Methods*. Ninety allogeneic HSCT patients, after transplant, were randomized to receive ISO-principle (i.e., mood matching) based music therapy (MT; *n* = 29), unstructured music (UM; *n* = 30), or usual care (UC; *n* = 31) for four weeks. The ISO principle posits that patients may shift their mood from one state to another by listening to music that is “equal to” the individual's initial mood state and subsequently listening to music selections that gradually shift in tempo and mood to match the patient's desired disposition. Participants in MT and UM groups developed two audio CDs to help them feel more relaxed and energized and were instructed to use the CDs to improve their mood as needed. Short-term effects on mood and long-term effects on QOL were examined. *Results*. MT and UM participants reported improved mood immediately after listening to CDs; the within-group effect was greater for UM participants compared to MT participants. Participant group was not associated with long-term QOL outcomes. *Conclusions*. Music listening improves mood acutely but was not associated with long-term benefits in this study.

## 1. Introduction

Allogeneic hematopoietic stem cell transplantation (HSCT) is used to treat a variety of malignant diseases. The procedure is regarded as one of the most difficult oncologic interventions due to the common and intense side effects of high dose chemotherapy and graft-versus-host disease such as organ toxicity (e.g., pulmonary and cardiac), osteoporosis, infection, cataracts, and infertility [1–8]. Not surprisingly, HSCT has been associated with diminished quality of life (QOL), especially in the first 100 days after transplant period [2–5]. The period of lowest white blood cell count, nadir, which typically occurs within the first 30 days after transplant, has been identified as the time when patients report the greatest symptom distress [9] although patients may report symptom distress for as long as 3–5 years after transplant [10]. The acute complications of HSCT may prevent patients from participating in common symptom management interventions [6]. Thus, finding effective methods to alleviate distress and improve coping skills and emotional well-being may improve post-transplant QOL as well as reduce symptom distress [2, 7, 8, 11].

Music therapy, which requires minimal physical exertion, may be an ideal intervention for helping HSCT patients manage their emotions and control stress. For the purpose of our study, it is important to draw a distinction between the passive and active use of music as an intervention. Passive music listening often involves listening to music without a music therapist, while active music therapy interventions involve being engaged with a music therapist influencing the listening experience towards a therapeutic, and sometimes insight-oriented, objective [12]. Music therapy has, with varied success, been used to alleviate a broad range of cancer-related symptoms, such as sleep difficulties, pain, anxiety, depression, fatigue, and nausea, and to increase coping skills and spiritual well-being [13–17]. A recent systematic review of 30 trials examining the effects of music interventions for adult cancer patients found that music interventions (both passive and active) were consistently associated with improvements in anxiety, mood, and pain and less consistently associated with improvements in fatigue and physical functioning [18]. To date, only two studies examined the effect of a music intervention for adult HSCT patients and suggest that music therapy may lower anxiety, depression, and mood disturbance [19], as well as increase psychological well-being and physical comfort [20]. These interventions were individualized, involving both passive music listening and active music creation, making it difficult to determine the mechanisms by which these music interventions led to beneficial effects. Studies examining manualized music therapy interventions would enable mechanisms of action to be identified and would allow for better replication and dissemination.

The ISO principle is a music therapy strategy that lends itself to a standardized treatment but has yet to be tested in a controlled trial. This principle was first put forth by psychiatrist Dr. Ira Altshuler in 1945 as a method of assisting psychiatric patients in shifting their mood from depressed to energized or from anxious to relaxed [21, 22]. The “ISO” (Greek for “equal”) principle involves beginning a music therapy session by listening to music that is “equal to” the individual's initial mood state and subsequently listening to music selections that gradually shift in tempo and mood to match the patient's desired disposition. For example, a patient feeling depressed may begin by listening to music with slow tempo that matches their mood. Throughout the session, the patient would listen to music selections that gradually increase in tempo and become closer to their desired mood (i.e., energized/joyful). A music therapy intervention based on the ISO principle may be ideal for populations that would benefit from a mood management technique requiring little physical exertion that can be used in a variety of settings (e.g., hospital or home).

The primary aim of the present study was to assess short-term (immediately after music listening) effects of the ISO-principle music therapy intervention. Specifically, we hypothesized that HSCT patients in the ISO-principle-based music therapy group (MT) would report greater improvements in mood immediately after listening to their music playlists compared to individuals in the unstructured music listening group (UM). The secondary aim was to assess the long-term (1 week and 1 month after intervention) effects of the ISO-principle music therapy intervention with the hypothesis that the MT group would, in turn, have better outcomes compared to the UC group 1 week and 1 month after the intervention.

## 2. Methods

### 2.1. Patients

Ninety patients, age 18 or older, who had undergone allogeneic HSCT and signed a consent less than 70 days earlier at MD Anderson Cancer Center between January 2004 and May 2005 were recruited for the study. For inclusion in the study, patients needed to be ready to transition from inpatient to outpatient status, or already an outpatient but yet still and within the first 100 days of transplant. Patients who had a known psychotic disorder were excluded from the study. Each patient provided written informed consent, and the study was approved by the Surveillance Committee for the Protection of Human Subjects.

### 2.2. Intervention

Patients were randomly assigned to one of three groups: (1) ISO-principle music therapy (MT) group, (2) unstructured music (UM) group, and (3) usual care (UC) control group. Patients in the MT group met with a music therapist (one board certified music therapist) for four 50-minute sessions. In the first session participants were introduced to the ISO principle and worked in collaboration with the music therapist to select 15 tracks from a large database that transitioned in tempo from *allegro* (120–168 beats per minute (BPM)) to *largo* (40–60 BMP) and in mood from anxious/tense to relaxed. In the second session, participants chose 15 tracks that transitioned in tempo from *largo* (40–60 BMP) to *allegro* (120–168 BMP) and in mood from sad/depressed to energized/joyful. Using these tracks, the music therapist created two 30-minute CDs: the first was designed to transition the patient from an anxious/tense state to a relaxed state and the second was designed to transition the patient from a sad/depressed state to an energized state. In session 3 participants reviewed the two CDs and made any necessary changes, and in session 4 participants chose one of their CDs and listened to it in its entirety.

Patients in the UM group met with a licensed mental health therapist (one therapist) for four 50-minute sessions. During session 1 they discussed the use of music to modify mood, listened to different music tracks, and selected 15 music tracks from the same database as the MT group that made them feel relaxed. In session 2, patients selected 15 music tracks that made them feel energized. The tracks were organized into two 30-minute CDs (one including relaxing songs and the second including energizing songs) based on personal preference with little input from the therapist. In session 3 participants reviewed the two CDs and made any necessary changes, and in session 4 participants chose one of their CDs and listened to it in its entirety.

The difference in the MT and UM groups was only that the tempo of the music as measured by BPM was modulated from fast to slow (relaxing CD) or slow to fast (energizing CD) in the MT group, while the BPM was not taken into consideration in the UM group's CDs. Patients in both groups were instructed to listen to their CDs to modify their mood as needed and were asked to record their mood before and after listening to their CDs in a listening log.

Patients in the UC group had no musical intervention, did not meet with a therapist, and received care as usual.

### 2.3. Procedures

At baseline, patients completed a 30-minute battery of questionnaires and patients' blood samples were drawn for future assay (to be reported in a subsequent manuscript). The patients were then randomly assigned to one of the three groups. MT and UM participants completed assessments of mood before and after their final music therapy session. Our rationale for analyzing pre- and postsession data is based on the idea that, if proven effective, patients in acute situations could utilize music therapy interventions for situational relief of symptoms linked to medical procedures [23]. All patients completed the mood and QOL measures at the 1-week and 1-month follow-up. All follow-up assessments were conducted within 100 days of HSCT. Patients' demographic and clinical data were extracted from medical records.

### 2.4. Measures

Changes in mood were assessed using the short form of the Profile of Mood States (POMS-SF) [24]. The POMS-SF, which is commonly used in cancer research, is a 37-item mood adjective checklist containing six subscales: tension-anxiety, depression-dejection, anger-hostility, vigor, fatigue, and confusion-bewilderment. To reduce the number of analyses, the total mood disturbance score is reported with high scores representing worse mood disturbance.

Health-related QOL was assessed using the Functional Assessment of Cancer Therapy-General (FACT-G) and the Bone Marrow Transplant (FACT-BMT) subscale. The FACT-G yields an overall QOL score and assesses four subscales: physical well-being, social/family well-being, emotional well-being, and functional well-being [25]. The BMT subscale is designed to assess QOL issues particularly relevant to patients who have undergone a HSCT. The FACT-G total score and BMT score are reported with higher scores representing better QOL and fewer symptoms.

Cancer-related symptoms were assessed using the MD Anderson Symptom Inventory (MDASI) which evaluates 13 symptoms that are common across all cancer diagnoses and treatments and also evaluates 6 functional areas in which the symptoms may have interfered [26]. Patients rate the intensity of each symptom and amount of interference they have experienced on a scale from 0 to 10. An average score was calculated creating a possible range of 0–10.

Participants were asked to complete a short music-listening log each time they used their CDs for 1 month after the intervention. Participants rated their level of relaxation/tension on a scale from 1 (extremely relaxed) to 10 (extremely tense) and their level of happiness/sadness on a scale from 1 (extremely happy) to 10 (extremely sad) before and after listening to their CDs. They also recorded which CD they listened to (relaxing or energizing) and the number of minutes that they listened.

### 2.5. Statistical Analysis

We used chi-square test and analysis of variance (ANOVA) to examine possible group differences in baseline demographic, medical, and self-report psychosocial variables. We used ANOVA to determine if MT and UM participants differed in the frequency and duration that they reported listening to their CDs. Using ANOVA, we examined whether demographic or medical variables were associated with self-report psychosocial outcome variables to determine relevant covariates. Group comparisons of the self-report psychosocial measures were performed by regressing the assessments for each measure on time, group assignment, the group by time interaction, and the respective psychosocial baseline measure using general linear mixed model regression analyses (LMM). Demographic and medical variables found to have an association with the outcome measure were included as covariates in these analyses. The intercept was treated as random, and the group effect was treated as a classification variable using class statement in PROC MIXED procedure. The analyses of short-term outcomes based on specific CDs with separate analyses, using LMM, included only the MT and UM groups, with the UM group as the reference group. In the analyses of long-term outcomes, the UC group was the reference group. We treated time as a classified variable using class statement and the last time point in each model was the reference time point. As an exploratory analysis, we also used LLM to examine changes in psychosocial outcome measures over time (from baseline to 1 month after intervention). We examined if there was an association between the use of the CDs after the training sessions and outcomes at 1 week and 1 month. The SAS statistical software program (version 9.2; SAS, Cary, NC, USA) was used to perform all analyses. Using G*Power [27], it was determined that a sample size of 21 patients for each treatment group would enable us to detect a treatment effect equal to 0.50 standard deviations with 95% confidence with 80% power. Anticipating attrition of 15% during the study, a total of 90 participants were recruited.

## 3. Results

### 3.1. Patient Characteristics

Ninety individuals completed baseline data (MT = 29, UM = 30, and UC = 31). Fifty-two percent of the participants were women, 66% married, and 66% of Caucasian ethnicity, with a mean age of 44.3 years. A variety of cancer diagnoses were represented, with the most common being leukemia (71%). Most patients received transplant cells from matched related donors (57%) and most donor cells were from peripheral blood (59%). Forty-two percent of patients experienced graft-versus-host disease (GVHD), and 53% had experienced a reactivation of cytomegalovirus (CMV). There were no group differences in demographic, medical, and baseline psychosocial characteristics (Table 1). POMS-SF ratings before and after session 4 were obtained for 97% of participants (MT = 28 and UM = 29) and mood ratings before and after listening to CDs during follow-up were obtained from 85% of participants (MT = 24 and UM = 26). There were no significant group differences in loss to follow-up. The mean number of days since transplant was higher for those who dropped out of the study by the 1-month follow-up compared to those who did not drop out of the study (34.5, SD = 15.20 versus 25.24, SD = 7.52). Otherwise, the demographic, medical, and baseline psychosocial variables did not differ between patients who did and did not drop out of the study by the 1-month follow-up.

### 3.2. Adherence and Feasibility

There were no differences between MT and UM groups in their reported use of the CDs at 1-month follow-up. Patient reports of the frequency or duration of CD use were not related to study outcomes. Additionally, there was no difference in frequency or duration of CD use between the two types of CDs (relaxing versus energizing) within the MT and UM groups. Participants listened to music CDs with an average of 9.90 (SD = 6.86) times for 38.87 (SD = 43.58) minutes each time during the 1-month follow-up. There were no differences between MT and UM groups in any report of satisfaction.

### 3.3. Demographic and Medical Variables Associated with Psychosocial Outcomes

Donor-recipient match status, CMV reactivation status, graft-versus-host disease (GVHD) status, time since transplant, and sex were not associated with any short- or long-term psychosocial outcomes. Diagnosis was associated with FACT-G (*P* < 0.01) and BMT (*P* = 0.02) scores at 1-week follow-up and BMT (*P* = 0.01) and POMS-SF (*P* = 0.01) scores at 1-month follow-up. Lymphoma patients reported higher general QOL compared to patients with leukemia or other diagnoses (i.e., renal, breast, or ovarian cancer and aplastic anemia) at 1-week follow-up (*P*'s < 0.01); however, patients diagnosed with either lymphoma or leukemia tended to report higher BMT-specific QOL compared to patients with other diagnoses (QOL at 1-week and 1-month follow-up; *P*'s < 0.05). Additionally, patients diagnosed with leukemia tended to report lower mood disturbance and fewer symptoms compared to patients with lymphoma or other diagnoses at 1-month follow-up (*P*'s < 0.01). Graft type (peripheral blood, bone marrow, or umbilical cord/placenta) was associated with MDASI at 1-week follow-up (*P* = 0.05) and FACT-G at 1-month (*P* = 0.04) follow-up. Specifically, individuals who received cells derived from umbilical cord/placenta reported poorer QOL and greater symptoms than those with cells derived from marrow or peripheral blood (*P*'s < 0.05). Furthermore, ethnicity was associated with POMS-SF, FACT-G, BMT, and MDASI scores at 1-week follow-up (*P*'s < 0.03). Non-Hispanic white participants reported better general and BMT-related QOL compared to Hispanic participants and lower MDASI scores compared to African American participants (*P*'s < 0.05). Hispanic participants reported higher POMS-SF scores compared to non-Hispanic white and African American participants (*P*'s < 0.01). Lastly, age was associated with POMS-SF and MDASI scores at 1-month follow-up, with older participants reporting greater mood disturbance and symptoms (*P*'s < 0.05). Thus, diagnosis, donor cell type, ethnicity, and age were entered as covariates in analyses examining group differences in self-reported short- and long-term outcome measures.

### 3.4. Short-Term Effects

**Figure 1:**
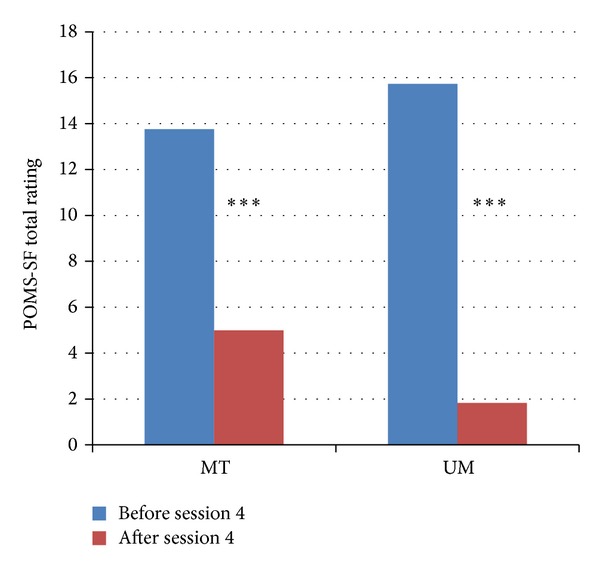
Least squared means of mood ratings before and after final music session. All means are adjusted for diagnosis, donor cell type, ethnicity, age, and baseline POMS-SF total score. POMS-SF scores were significantly reduced from before to after final music session (session 4) regardless of group. ***P < 0.0001. MT: music therapy; UM: unstructured music.

The first short-term outcome (POMS-SF total score before and after the final music session) was examined using LMM covarying for baseline POMS score, diagnosis, donor cell type, ethnicity, and age. Results revealed a significant effect of time (i.e., before versus after session 4; *P* < 0.0001), with individuals reporting reduced mood disturbance after their final music session compared to their mood prior to the session (see Figure 1 for least squared means). There was no effect of group or group-by-time interaction on POMS-SF scores after the final music session.

**Figure 2:**
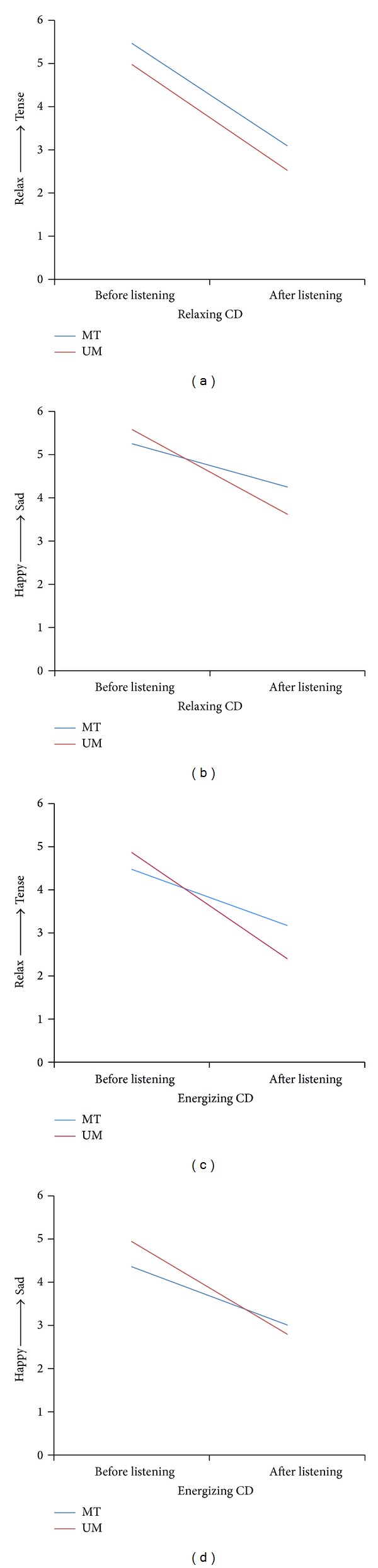
Least squared means of mood ratings before and after listening to CDs outside of session. All means are adjusted for diagnosis, donor cell type, ethnicity, age, and number of minutes the individual listened to the respective CD. All means improved from before to after listening (*P* < 0.0001). A significant group-by-time interaction effect was found in Figures (b)–(d) (*P* < 0.01), though post hoc *t*-test did not reveal significant group differences before or after listening (*P* > 0.07). Mood assessed by rating relaxation/tension on a 1 (extremely relaxed) to 10 (extremely tense) scale and happiness/sadness on a 1 (extremely happy) to 10 (extremely sad) scale. MT: music therapy; UM: unstructured music.

The second short-term outcome (mood before and after listening to CDs at the 1-month follow-up) was examined using LMM, covarying for diagnosis, donor cell type, ethnicity, age, and the number of minutes that patients listened to their CDs. Results did not reveal a significant effect of group on relaxation/tension or happiness/sadness ratings. Analyses did indicate a significant effect for time (i.e., before versus after listening) for both CDs (relaxing and energizing) on ratings of relaxation/tension and happiness/sadness (all *P*'s < 0.0001; see Figures 2(a)–2(d) for least squared means), such that mood was rated as more relaxed and happier after listening to the CDs. Though there was no group-by-time interaction effect for the relaxing CD on relaxation/tension ratings (Figure 2(a)), significant group-by-time interaction effects were found for the relaxing CD on happiness/sadness ratings (*P* < 0.01; Figure 2(b)) and for the energizing CD on relaxation/tension (*P* < 0.001; Figure 2(c)) and happiness/sadness (*P* < 0.01; Figure 2(d)) ratings. However, post hoc analysis comparing the MT and UM groups at each time point (i.e., before and after listening to music) did not reveal any significant group differences in mood ratings before or after listening to either CD (all *P*'s > 0.07).

### 3.5. Long-Term Effects

LMM analyses, controlling for the baseline score of the outcome variables and covarying for diagnosis, donor cell type, ethnicity, and age, did not reveal a significant effect of group, time, or group-by-time interaction on FACT-G, BMT, POMS-SF, or MDASI scores (Table 2). Exploratory LLM analyses were then conducted to examine the effect of time (from baseline to 1 month after intervention) on psychosocial outcome measures for all groups. Group, diagnosis, donor cell type, ethnicity, and age were entered as covariates. Patients reported improved FACT-G (*P* = 0.001), BMT (*P* = 0.001), POMS-SF (*P* < 0.0001), and MDASI (*P* = 0.01) scores over time (from baseline to 1 month after intervention).

## 4. Discussion

The findings of the present study did not support our hypothesis that listening to CDs that follow the ISO principle would result in greater acute improvement in mood compared to listening to unstructured CDs designed by participants to relax or energize. Indeed, participants (regardless of group) reported improved mood immediately after listening to their playlists, both during session 4 and the 1-month follow-up. Interestingly, participants listening to unstructured playlists reported slightly greater improvements in mood immediately after listening to their CDs during the 1-month follow-up compared to participants whose playlists followed the ISO principle, with tracks gradually transitioning in tempo and mood from anxious to relaxed or from depressed to energized.

There are several possible reasons for why the ISO-based music therapy intervention did not result in greater improvements in acute mood compared to unstructured music listening. First, there is little research on the ideal parameters to use when implementing ISO principle (e.g., length of time between musical track transitions, etc.), making it possible that a different implementation of the ISO principle may have been more effective at improving mood than the method used in the present study. Additionally, there are no studies examining the ISO principle in a medical population, and in fact the theory was developed to assist psychiatric inpatients in managing their mood. Thus, it is possible that patients without clinical mood disorders derive greater benefit from listening to consistently relaxing or energizing music, rather than listening to playlists transitioning from one music type to another. In addition, patients in the MT were constrained to listening to music therapy tracks in an order that followed the ISO principle, whereas the UM group had the freedom to choose their songs in any order. The imposition of a structured order of music may have taken away personal preference and may have accounted for the improvement in the UM group.

The present study did not support our secondary hypothesis that individuals in ISO principle music therapy intervention would derive greater long-term benefit than patients in an unstructured music listening or a usual care control group or that those in an unstructured music listening group would report better QOL than those receiving usual care. Moreover, independent of group assignment, individuals reported significant improvements in QOL from baseline to 1 month after intervention, echoing previous research suggesting that QOL continues to improve during the first 100 days after transplant [1, 2]. There are several possible reasons for the lack of benefit. First, participants reported that they listened to the CDs infrequently (only nine times on average during the one-month period after the intervention). It is possible that, with more frequent use, a long-term effect of music listening on QOL may have been found. This may be especially important for the MT group, as the length of exposure to a particular beat may determine the brain's ability to entrain, or to assume the rhythm of the musical selection, which then permits the progressive transition from one state to another described in the ISO principle [28]. Short presentations of as little as four minutes (the maximum length of most music tracks) of binaural beats may not be sufficient to alter patient's physiological vigilance, thereby making it impossible to fully “transition” from one mood state to another [29]. Second, the time at which the intervention was conducted may be another reason for the lack of long-term intervention effect. Patients were recruited at an average of 28 days after transplant, after much of their recovery had already taken place [30]. It is possible that delivery of our intervention at an earlier time point could have significantly affected patient outcome. Relatedly, it is important to note the potential for a “ceiling effect” for the psychosocial outcomes. This ceiling effect may have left little room for improvement, meaning that it is possible that patients who volunteered to participate in this study may have been doing relatively well physically and emotionally compared to their more impaired, possibly hospitalized, transplant patient peers. In fact, the FACT-G means reported at baseline in the present study (*M* = 73.78; SD = 14.40) were within 1 standard deviation of the established norms for the general United States adult population (*M* = 80.1; SD = 18.1) [31]. However, Sherman and colleagues report a mean FACT-G score for a post-HSCT sample that is comparable to ours (*M* = 71.31; SD = 15.37), suggesting that our sample may have been an accurate representation of post-BMT patients [32].

Though music therapy was not related to study outcomes, several medical and demographic variables were associated with QOL, mood, and cancer-related symptoms. First, the present study indicates that older patients experience greater mood disturbance following HSCT. Similarly, Niederbacher et al. [33] also found that age was associated with mood disturbance, although their study suggests that middle age patients may experience the greatest distress compared to the oldest and youngest patient groups in some measures. Secondly, unlike previous research that reported no association between ethnicity and QOL [34], we found that non-Hispanic white patients tend to report less severe symptoms and better QOL following HSCT compared to patients of ethnic minorities and that African American patients may experience less mood disturbances following HSCT compared to Hispanic participants. Similar to findings from previous studies [35], the present study suggests that diagnosis may be related to QOL following HSCT, with patients diagnosed with lymphoma experiencing better posttransplant QOL compared to patients with other diagnoses. Lastly, present findings suggest that individuals receiving transplant cells derived from umbilical cord/placenta may experience poorer QOL and greater symptoms than those whose grafts are derived from bone marrow or peripheral blood. Though this association has not been previously reported, many studies have excluded patients receiving cells derived from umbilical cord/placenta, likely due to their relatively small numbers [36, 37].

There are several limitations in this study. Though there was no gender effect, it is important to note that the therapist working with the MT group was male and the therapist working with the UM group was female. Using one therapist to treat both intervention and active control group introduces a potential unintentional bias in how they work with the groups. Additionally, there was relatively high attrition (25%) at 1 month, and thus the results may not be generalizable. Lastly, the majority of participants were white, non-Hispanic, married, and highly educated. Thus, future research is needed to test the generalizability of these findings to more diverse populations.

Although the present study did not reveal long-term QOL group differences in response to two music therapy interventions, it did show that listening to music identified as “relaxing” and “energizing” results in acute improvements in mood. Furthermore, the present study suggests that this effect may be slightly greater when individuals listen to unstructured playlists consisting of music they identify as “relaxing” or “energizing” compared to playlists designed to gradually transition to “relaxing” or energizing.” Those wishing to further investigate the effects of the ISO principle music therapy technique should consider ensuring that the music choice options are all patient-derived and then assembled by the therapist based on BPM. This will allow the greatest connection for the patients. Future studies examining music therapy in this population should focus on methods to increase patient compliance to music listening to optimize long-term benefits.

## Figures and Tables

**Table 1:** Patient demographic and medical data.

Characteristic	Music therapy *n* = 29	Unstructured music *n* = 30	Usual care *n* = 31
Women *N* (%)	14 (48)	12 (40)	21 (68)
Mean age (SD)	43.72 (14.63)	44.07 (13.56)	45.19 (11.04)
Ethnicity *N* (%)			
White	20 (69)	20 (67)	19 (61)
African American	0 (0)	3 (10)	4 (13)
Hispanic/Latino	4 (14)	4 (13)	3 (10)
Asian	1 (3)	1 (3)	2 (6)
Other	4 (14)	2 (7)	3 (10)
Marriage status *N* (%)			
Married	20 (69)	22 (73)	18 (58)
Divorced	2 (7)	1 (3)	2 (6)
Never married	5 (17)	2 (7)	6 (19)
Declined to answer	2 (7)	5 (17)	5 (16)
Education *N* (%)			
High school diploma	3 (10)	6 (20)	2 (6)
Some college	12 (41)	11 (37)	12 (39)
College degree	7 (24)	7 (23)	10 (32)
Graduate degree	4 (14)	4 (13)	3 (10)
Declined to answer	2 (7)	2 (7)	4 (13)
Diagnosis *N* (%)			
Leukemia	18 (62)	20 (67)	19 (70)
Lymphoma	6 (21)	2 (7)	5 (19)
Other	5 (17)	8 (27)	7 (11)
Donor match status			
Matched related	18 (62)	16 (53)	17 (55)
Matched unrelated	8 (28)	11 (37)	9 (29)
Unmatched unrelated	1 (3)	1 (3)	1 (3)
Unknown	2 (7)	2 (7)	4 (13)
Donor cell type *N* (%)			
Peripheral blood	18 (62)	17 (57)	18 (58)
Bone marrow	9 (31)	11 (37)	8 (26)
Umbilical cord/placenta	0 (0)	0 (0)	1 (3)
Unknown	2 (7)	2 (7)	2 (6)
Baseline GVHD *N* (%)			
None	19 (66)	15 (50)	20 (65)
Grade 1	5 (17)	6 (20)	5 (16)
Grade 2	4 (14)	6 (20)	4 (13)
Grade 3 or 4	1 (3)	3 (10)	2 (6)
Baseline CMVR *N* (%) yes	13 (45)	17 (57)	18 (58)
Mean days since transplant (SD)	25.52 (10.10)	26.96 (9.40)	30.04 (12.10)

GVHD: graph-versus-host disease; CMVR: cytomegalovirus reactivation.

**Table 2:** Mean Functional Assessment of Cancer Therapy-General (FACT-G), Bone Marrow Transplant subscale (BMT), Profile of Mood States-Short Form (POMS-SF), and MD Anderson Symptom Inventory (MDASI) Scores.

	Baseline *N* = 90 Mean (SD)	1 week *N* = 81 Mean (SD)	1 month *N* = 68 Mean (SD)
FACT-G			
MT	76.70 (14.79)	77.92 (12.00)	82.81 (15.77)
PM	70.05 (17.88)	74.84 (13.70)	75.76 (13.22)
UC	71.98 (15.97)	73.12 (13.97)	75.85 (16.00)
BMT			
MT	26.29 (5.56)	27.57 (5.45)	28.41 (6.28)
PM	23.20 (6.47)	24.99 (5.48)	25.51 (4.30)
UC	22.82 (6.14)	24.00 (5.80)	24.92 (5.96)
POMS-SF			
MT	15.31 (19.975)	8.59 (19.12)	5.70 (24.92)
PM	14.30 (16.397)	5.11 (14.53)	6.38 (20.02)
UC	16.06 (23.123)	9.32 (20.21)	8.29 (15.29)
MDASI			
MT	2.31 (1.71)	2.08 (1.62)	1.92 (2.19)
PM	3.05 (1.73)	2.47 (1.14)	2.15 (1.20)
UC	2.69 (1.74)	2.73 (1.90)	2.31 (1.41)

SD: standard deviation; MT: music therapy; PM: unstructured music; UC: usual care.
